# Electronic Health Records in Specialized Pediatric Palliative Care: A Qualitative Needs Assessment among Professionals Experienced and Inexperienced in Electronic Documentation

**DOI:** 10.3390/children8030249

**Published:** 2021-03-23

**Authors:** Dorothee Meyer, Sven Kernebeck, Theresa Sophie Busse, Jan Ehlers, Julia Wager, Boris Zernikow, Larissa Alice Dreier

**Affiliations:** 1 PedScience Research Institute, 45711 Datteln, Germany; j.wager@pedscience.de (J.W.); b.zernikow@kinderklinik-datteln.de (B.Z.); l.dreier@pedscience.de (L.A.D.); 2 Department of Children’s Pain Therapy and Pediatric Palliative Care, Faculty of Health, School of Medicine, Witten/Herdecke University, 58448 Witten, Germany; 3 Chair of Didactics and Educational Research in Health Science, Faculty of Health, Witten/Herdecke University, 58448 Witten, Germany; sven.kernebeck@uni-wh.de (S.K.); theresa.busse@uni-wh.de (T.S.B.); jan.ehlers@uni-wh.de (J.E.); 4 Pediatric Palliative Care Centre, Children’s and Adolescents’ Hospital, 45711 Datteln, Germany

**Keywords:** palliative care, pediatrics, electronic health records, usability, demand analysis

## Abstract

Background: Currently, to the best of our knowledge, no findings exist concerning the needs of professionals in specialized pediatric palliative care (PPC) regarding electronic health records (EHRs). Several studies have highlighted benefits concerning the use of EHRs in pediatrics. However, usability is strongly affected by the degree of adaptivity to the context of application. The aim of this study is to examine the needs of professionals concerning an EHR in the specialized PPC inpatient and outpatient settings. Methods: A qualitative research design was chosen to address the complex aspects of user demands. Focus group interviews and semi-structured one-on-one interviews were conducted with PPC professionals. *N* = 23 participants from inpatient and *N* = 11 participants from outpatient settings of specialized PPC representing various professions took part in the study. Results: The findings could be grouped into four categories: (1) attitude towards the current methods of documentation, (2) attitude towards electronic documentation in general, (3) general requirements for an EHR, and (4) content requirements for an EHR. Conclusions: Professionals in specialized PPC expect and experience many benefits of using electronic documentation. Their requirements for an EHR for inpatient and outpatient settings of PPC are largely consistent with EHRs for pediatrics. However, individual specifications and adaptations are necessary for this particular setting.

## 1. Introduction

The use of electronic health records (EHRs) in healthcare systems is increasingly common and, often, even obligatory [1,2,3]. Although electronic documentation is meant to facilitate and support health care services, problems related to usability and patient safety have been identified in several studies [2,4,5]. Usability is understood as the “extent to which a product can be used by specified users to achieve specified goals with effectiveness, efficiency, and satisfaction in a specified context of use” [6] (p. 144). According to a recent review, poor EHR usability is a contributor to physician dissatisfaction; a direct relationship between EHR usability and physician burnout may exist [5]. Ratwani et al. (2018) analyzed 9000 patient safety reports in pediatric settings between 2012–2017 that were likely related to EHR use [2]. Of those reports, 3243 (36%) had usability issues associated with a medication event, and 609 of the 3243 reports (19%) may have caused patient harm. The authors identified system feedback and visual display as the most common usability challenges and improper dosing as the most common medication error. EHR-embedded clinical decision support tools for drug dosing could provide assistance in this regard [7]. The results of a national usability survey of hospital information technology in Germany showed that specialized information systems with well-defined functionality scored better than clinical information systems in general [8]. However, Leu et al. (2012) illustrated that pediatricians have considerable doubts about finding systems that meet their specific needs [9].

In 2020, the American Academy of Pediatrics published a policy statement [10] and an accompanying technical report [3] to establish the standards of an EHR for pediatrics by evaluating the unique aspects of clinical documentation for pediatric care in contrast to adult care. The technical report offered several general requirements concerning content and functionality. Nevertheless—and this is supported by the aforementioned findings—the specific requirements of a setting should be considered in addition to general suggestions.

A highly complex field of pediatrics is pediatric palliative care (PPC). PPC supports children and adolescents with “life-limiting and life-threatening conditions, from the point of diagnosis or recognition throughout the child’s life and death” [11] (p. 9) and their families. Most patients in PPC have severe neurological and physical impairments due to a large variety of rare diseases [12,13,14]. They suffer a broad spectrum of interrelated symptoms [15] and are in need of a plethora of medication and aids [12,16].

PPC can be provided on different levels, categorized by the grade of specialization. Specialist palliative care teams represent the highest grade of specialization. They are implemented in hospitals, hospices, or communities and are typically staffed with physicians, nurses, psychologists, or social workers with specialty training in PPC [11]. Specialized outpatient PPC teams (SOPPC) offer home care with a medical focus [12]. Inpatient care in specialized PPC units (PPCU) may be required in case of complications, symptom escalations at home, or when outpatient PPC structures lack sufficient resources to address complex symptom constellations [12,17].

Due to the often long-term and complex care for patients and their families, a supporting documentation system that meets the special demands of this setting is vital. As PPC differs from adult palliative care in several ways [11], EHRs used in this setting may not be able to meet the special documentation demands in PPC.

Several studies have highlighted user participation in the development of EHRs as a crucial factor for system success, i.e., user acceptance and usability [18,19,20,21]. To our knowledge, no studies considering the special demands of providers concerning electronic documentation in specialized PPC are available. The aim of this study is therefore to analyze the user demands of an EHR for specialized inpatient and outpatient PPC as the basis for developing a PPC EHR that ensures usability for professionals and enhances the care of a highly vulnerable and complex patient population.

## 2. Materials and Methods

### 2.1. Design

In order to explore users’ requirements and preferences of inpatient and outpatient EHRs in specialized PPC, a qualitative approach to the content analysis [22] was chosen. Where routines allowed, we conducted profession-specific focus group interviews using a semi-structured guideline. When participants could not set up focus group interviews, we conducted semi-structured one-on-one interviews.

The data collected in this study were part of the project ELSA-PP (Electronic cross-sectoral health record for pediatric palliative care) funded by the European Regional Development Fund (ERDF); grant reference: EFRE-0801385.

### 2.2. Participants and Recruitment

Healthcare professionals composed of physicians, nurses, and psychosocial staff from one PPCU and three SOPPC teams in Germany, as well as secretaries from a PPCU (nonclinicians), were included. PPCU healthcare professionals use paper health records for documentation. PPCU secretaries were included, as they review records for completeness for the purpose of accountability. The SOPPC teams were already utilizing a web-based EHR that was initially developed for adult palliative care patients.

Participants obtained detailed verbal and written study information, a sociodemographic questionnaire, and provided written informed consent prior to participation. All participants received monetary compensation.

In the inpatient setting, five focus group interviews with 23 participants were conducted. Focus group interviews lasted between 39 and 79 min. In the outpatient setting, one focus group interview and nine one-on-one interviews were conducted with 11 participants in total. Altogether, the outpatient one-on-one interviews and focus group interviews lasted between 25 and 58 min. Participants’ characteristics are shown in Table 1, separated by setting. Nineteen (*N* = 23) inpatient participants and nine (*N* = 11) outpatient participants completed the sociodemographic questionnaire. Seven inpatient participants reported experience in the professional use of EHRs. All nine participants from the outpatient setting had experience with documentation in EHRs, but only seven of them provided information on their years of experience with EHRs.

### 2.3. Data Collection

Data collection took place between June and August 2019. Focus group interviews and one-on-one interviews were conducted by members of the research team (L.D., D.M., T.S.B., and S.K.) with a proficiency in qualitative research. Beforehand, interview guidelines were created through discussion and consensus among the research team. The final versions included the following key questions:

- Which are the greatest strengths and weak points of the currently used type of record (paper record/EHR for adult palliative care patients)?
- What are your wishes for and concerns about EHRs in general?
- We would like to go through the currently used paper records/EHRs for adult palliative care patients:
  ◦ Which components do you generally like? And why?
  ◦ Which components would you like to see incorporated into the new system? And why?
  ◦ Which components would you like to be revised before being incorporated into the new system, e.g., concerning content, structure, and design?
  ◦ Which new components would you like to add to the new system? What would they look like specifically?

Focus group interviews and one-on-one interviews were audiotaped and subsequently transcribed verbatim.

### 2.4. Data Analysis

Data were analyzed by means of structuring content analysis [22] using MAXQDA V.18. Two researchers (T.S.B. and S.K.) independently read and coded transcripts deductively and inductively. The deductive approach was used to systematically structure the data based on the interview guideline. The inductive approach was used to identify patterns that emerged from the data. T.S.B. and S.K. reconciled the categories through discussion until a consensus was reached. To enable comparability between the inpatient and the outpatient settings, similar categories for both settings were defined. The coding system was then discussed among the research team until a consensus was reached. For this manuscript, several subcategories were synthesized for the purpose of a clear presentation (D.M). Participants’ original quotes were translated into English, and all participants were assigned pseudonyms using the following structure: “Focus Group (FG)/Interview (I)_Setting (PPCU/SOPPC)_Professional group_Number of FG/I: Transcript line number” (e.g., FG_PPCU_Nurse_5:12). If quotations contain statements of several participants, each individual is assigned a number (e.g., “P1”).

### 2.5. Ethical Approval

The study was conducted in accordance with the ethical principles specified in the Declaration of Helsinki. Ethical approval was obtained by the Ethics Committee of the Witten/Herdecke University, Witten, Germany (ID 35/2019).

## 3. Results

The transcript analysis revealed four main categories applicable to the inpatient and outpatient settings: (1) attitude towards the current methods of documentation, (2) attitude towards electronic documentation in general, (3) general requirements for an EHR, and (4) content requirements for an EHR. The inpatient setting comprised 18 subcategories, and the outpatient setting comprised 19 subcategories.

### 3.1. Attitude towards the Current Methods of Documentation

#### 3.1.1. Weak Points

According to the PPCU participants, a weak point of the current documentation is difficulty in quickly finding a paper record or specific document.

“I’m searching for a document and at first I think ‘oh no, it’s missing’, but then I see that it was misfiled.”

(FG_PPCU_Secretary_01:50-55)

Other weaknesses include documentation unrelated to the paper record (e.g., different calendars for admission planning and family meetings, task books for physicians and nurses, shift handover protocols, and patient flipcharts used for multi-professional team meetings). Problems with illegible documentation were also mentioned.

In contrast, participants from the SOPPC teams are used to working with a web-based adult EHR. They often experience connectivity issues, especially in rural areas. In addition to compromised data access, this leads to duplicate documentation.

“So, in [place] for example, it takes a very long time to boot, or in [place] I couldn’t access [the EHR; D.M.] because it took so long. When you need information quickly, that’s obviously difficult.”

(I_SOPPC_Nurse_02:83)

“Well, because it crashes from time to time, and then you get kicked out with only half of your document […] And that’s why, as a precaution, we always document in [word processing program; D.M.].”

(I_SOPPC_Psychosocial_01:55)

#### 3.1.2. Strengths

Strengths of the current state of documentation in a PPCU include the traceability of entries in the paper record based on a familiarity with colleagues’ handwriting, the ease of placing all relevant documents in the paper record, and habit-based manageability.

SOPPC participants specifically appreciated the accessibility from different locations, the availability of relevant contact details of involved suppliers, the file upload function, the task tracking tool, and the statistics generation feature (for billing purposes).

“In fact, the greatest strength is that it is web-based. That you can actually easily access the data stored there from anywhere. That means, on every car journey to the family, on every home visit to the families, that you always can find something quickly. Even if it is just the insurance number or whatever. I think that is the greatest strength.”

(I_SOPPC Nurse_03:13)

### 3.2. Attitude towards Electronic Documentation in General

#### 3.2.1. Expectations

PPCU participants anticipated that an EHR would be beneficial when readmitting patients, as it would allow easy access to former treatment records (e.g., assessments and care or nutrition plans).

“I think about having nightshift in the hospital […] in the middle of the night, an ambulance and an emergency physician arrive and say: ‘Here is a child known in your PPCU […]’, but they did not bring any documents from the child’s home. Then I don’t know the medication.”

(FG_PPCU_Physician_01:231-242)

Furthermore, PPCU participants mentioned that simultaneous documentation and reduced redundant documentation (for instance, concerning order management) would be useful features of an EHR.

PPCU secretaries expected a simplification of the workflow with medical accounting professionals due to the continuous accessibility of the EHRs.

“Perhaps the ladies of the coding department, they also browse through everything that could be relevant to them, what can still be billed. And if there are clear things where they just have to check up […] then this would accelerate the workflow; that there is a link, that they no longer have to search for everything they need […].”

(FG_PPCU_Secretary_01:173)

#### 3.2.2. Concerns

PPCU participants’ concerns about electronic documentation referred to system stability and data loss (e.g., program crashes), and, thus, to issues related to patient care and safety. Participants also expressed fears that low computer literacy could result in problems with electronic documentation, such as accidental content deletion, especially when people are used to handwritten documents.

SOPPC participants raised security concerns and pointed out the necessity to prevent data misuse.

Some SOPPC participants already working with an adult EHR expressed concerns about the potential implementation of a new system because of their familiarity with the structures and functionality of the current EHR. However, it was also noted that a fundamental adaptation to pediatrics is needed. Users criticized that the need for adaptation to the software currently in use was not sufficiently taken into account.

“To be honest, all of us in the team took it like this: ‘here comes something new again’. We have just managed to get used to something. I don’t know if the wish was that we needed something completely new. We would rather have wished that more of the wishes we had worked out together would gradually be adopted.”

(I_ SOPPC_Physician_05:38)

“My only fear is that all the things that […] we have brought up to certain people again and again are simply not taken seriously and are not going to be implemented, right? […] So, I think it would be desirable […] if the perspectives of various professional groups were taken seriously. And actually, would simply be checked for practicability and then, if necessary, integrated or not, if they are simply not practicable.”

(I_ SOPPC_Nurse_03:151)

### 3.3. General Requirements for an EHR

Participants mentioned many general requirements for an EHR (see Table 2). A detailed description and prototypical quotes for each requirement are provided in the text below.

#### 3.3.1. Different Data Entry Options

Participants from PPCU and SOPPC mentioned different tools for data entry that could facilitate electronic documentation, such as speech recognition software or documentation with ePens. These could be useful for professionals who are not familiar with typing (e.g., the ePens in family or patient meetings). Templates including checkboxes, dropdown menus, and spaces to enter discrete data could also aid documentation, but notwithstanding these tools, free text entries will remain necessary.

“[…] for us in nursing, some things cannot be well documented, in my opinion. Because there aren’t any structured forms behind it. If it somehow relates to wound management or tracheostomy care or in the direction of secretion management, there we still write a lot of running text. I think it would make it easier […] to have one short dataset to deposit [this information; D.M.].”

(I_SOPPC_Nurse_03:29)

#### 3.3.2. Clarity

Relating to clarity, PPCU professionals proposed that individualizable views would help pinpoint single parameters of interest, especially related to symptom observation.

“I think we will create sets […] such as a view for spastic and one for patients suffering from pain. But too many different views will become difficult. […] However, if these could be modified […].”

(FG_PPCU_Physician_01:415-421)

Furthermore, different professions highlighted that having both a daily and weekly view would enable a scalable overview of symptom progression.

“And especially in terminal care, there is so much going on […] that we would need to spread documentation space, so that everything we do could be documented.”

(FG_PPCU_Physician_01:305)

Moreover, there was a notable demand for filter functions based on search terms or data categories concerning special issues.

“If you are looking for something specific, the filters and search functions are not super convenient. It is sometimes a bit difficult to find exactly what you are looking for. Someone called a few weeks ago and said that it was not easy. So, things like that, they get you bogged down quickly because there are too many levels.”

(I_SOPPC_Physician_02:11)

#### 3.3.3. Integration of Multimedia Files

Another requirement from PPCU participants was the possibility to integrate multimedia files in an EHR, such as photo, audio, or video files concerning symptom documentation, documentation of therapeutic interactions, or tutorials for home care.

“We do that often; videos about seizures, agitation. Maybe about [social; D.M.] interactions.”

(FG_PPCU_Psychosocial_01:240)

#### 3.3.4. Interoperability

Interoperability was another need emphasized for usability by PPCU and SOPPC participants. Data should be exchangeable between wards of the hospital and the involved providers.

“Basically, you need a system–these are patients who come and go. Sometimes they are inpatient, sometimes outpatient, then they are x-rayed, then they have laboratory tests, then they have microbiological tests, then there are genetic findings, and everything has to be merged in a meaningful way. That would be very desirable in that it becomes superfluous to transfer patients from one system to the other just as it is superfluous to log into other systems.”

(I_SOPPC_Physician_04:85)

“[…] that a prescription is made here but ends up automatically–without requiring a fax or e-mail–at the relevant pharmacies.”

(I_SOPPC_Physician_05:63)

#### 3.3.5. Multi-Professional Documentation

PPCU participants expressed the need for multi-professional documentation that merges the relevant information from the different professions involved in the care process.

“We work together in a multi-professional team and speak often […], but somehow our documentation is so extremely separate from each other.”

(FG_PPCU_Psychosocial_01:31)

“Is there an option where all relevant information from all three professions could converge in one document? That would actually reflect it best. And where, perhaps, the least amount of information would be lost.”

(FG_PPCU_Psychosocial_01:258)

#### 3.3.6. Adaptability

Finally, participants from PPCU and SOPPC emphasized the importance of adaptability in an electronic system’s flexibility and customizable content.

“[…] how well can new systems or other documents, beyond a PDF, also be implemented? How flexible is the record when developing future documentation systems?”

(FG_PPCU_Physician_01:92)

### 3.4. Content Requirements for an EHR

Participants identified several specific content requirements for an EHR, from which nine content elements could be derived (Table 3). Detailed descriptions and prototypical quotes for each requirement are available in the text below.

#### 3.4.1. Individual Page with Patient’s Core Information

The SOPPC team participants desired an individual page, where patients’ core information (e.g., master data, genogram (detailed family relationships diagram [23], main problems, medical orders for life-sustaining treatment [24], medication, diagnoses, and admission reason) could quickly be assessed.

“It should note ‘guardian so and so’, contact person and phone number, and place of residence. […] I would actually put a genogram on it. You could also see diagnosis, name, place of residence, SOPPC, grade level, school. […] When you open that view, you have everything at a glance. And then, if you want to know more details you can search for healthcare providers, pharmacies, I don’t know, even speech therapy, occupational therapy.”

(I_SOPPC_Psychosocial_01:13)

#### 3.4.2. Basic Assessment Tool

From the PPCU professionals’ points of view, a basic assessment tool should be able to capture the medical, nursing, and psychosocial parameters on admission that are relevant for the heterogeneous group of PPC patients (e.g., patient’s social background, educational institutions, and relatives’ contact details). In particular, listing the patient’s abilities is highly relevant for severely disabled children who often develop special communication patterns.

“That means I simply need to extract a few other parameters for this patient group; how does he communicate, and so on […].”

(FG_PPCU_Physician_01:540)

“But, for example, you could also go into detail by saying something like ‘can distinguish light and dark’ or ‘turns his head when noticing light and dark’.”

(FG_PPCU_Psychosocial:119)

SOPPC participants also indicated the need for pediatric-specific categories, motivated by their experience with a primarily adult system. Information is needed, for example, on guardianship, social law and care benefits, degree of disability, outpatient children’s hospice services, nutrition, educational institutions, or living groups.

“I think, with all these templates, it´s still a little bit oriented towards an adult system. With regard to advance care planning, health care proxy, care proxy, who is entitled to care, for example, we write in the free text fields. That is not listed in detail.”

(I_SOPPC_Physician_05:48)

#### 3.4.3. Treatment Process

In addition, all participating professions from SOPPC and PPCU that are involved in patient care highlighted the need for a treatment process in which the main problems, treatment goals, interventions—including underlying decisions—and treatment outcomes can be documented in a traceable manner throughout the entire treatment period.

“[…] a process documentation, that shows–apart from daily documentation–a summary of the entire process. That would be an advantage […] in case of readmission, to understand why we made that decision at the time.”

(FG_PPCU_Physician_01:125)

“[…] What we always want is to have an overview; what are the symptoms? What are the orders? What has happened in terms of interventions? We accompany many families over many years, then they come for the second, third, fourth–whatever–time. So that one does not lose so much [information; D.M.] from the previous stays. But that you somehow have an overview, a kind of overview sheet, which is always immediately transferred to the next stay so that you can refer to it. Something clear, where the most important things are on it, so to speak. What one has is immediately presented again at the next stay.”

(FG_PPCU_Psychosocial_01:38)

“P3: We accomplish most symptom control via phone. We are on a home visit, hear about it, tell the parents to ‘do this and that’, and call […] one, two, three, five days later to hear whether the interventions […] had any effect […]. I would really like to have an overview for each child; these are the painful symptoms, this was once the leading symptom, we made these interventions and the symptoms improved. […]

P1: Yes, I think that’s good, too. The distressing symptoms and then which interventions were made.”

(FG_SOPPC_Nurse_01:31-32)

#### 3.4.4. Patient Chart

An EHR for inpatient PPC should include a patient chart in which different parameters like symptoms, positioning, medication, or vital signs are simultaneously displayed and, thus, can be put into context with each other. At the same time, this patient chart would also allow the different parameters within a group to be compared (e.g., interactions of different symptoms).

“P2: So, when a symptom is mentioned and the nurse says ‘today he has so much secretion again’, we really think on three levels. We think medically, […] we think, maybe he has an infection now. We think which nurse has not positioned him properly […] so that it can drain. Maybe inhalations have been forgotten. He got so upset because his mother went home and he is breathing stertorously with more secretions again. So, if this concern arises…

P8: This complexity […]

P2: Exactly, represents.”

(FG_PPCU_Physician_01:147-149)

“The documentation of observed behavior is often an issue. […] If you want to analyze agitation for instance, […] is it pain? […] Is it an interaction, boredom, whatever? That doesn´t work here.”

(FG_PPCU_Psychosocial_01:190-196)

In general, PPCU participants were oriented to the familiar paper record vital signs chart, while SOPPC participants noted that they lacked a documentation of symptoms, vital signs, or nursing reports comparable to the inpatient setting with paper documentation.

“We don’t even have any progress documentation right now [in the adult EHR; D.M.]. That means we just have a list of things, but no patient chart like in the inpatient settings. To be able to depict something over an entire day […]. that you can see one, two, three days next to each other […] so that you somehow have a short summary of the day above, perhaps including vital signs so that you get an overview of how the patient was, so to speak. I would find that quite useful.”

(I_SOPPC_Physician_02:25)

#### 3.4.5. Medication Tool

From PPCU and SOPPC physicians’ points of view, a medication tool that consists of a medication chart and calculation aid is essential. The medication chart should be adapted to the PPC needs and support physicians’ workflows. They suggested the possibility of sorting individual medications by dosage form or by therapeutic purpose. The documentation of the active ingredient and preparation name should be mandatory. Furthermore, medication orders should be able to be simply edited, such as by drag and drop. According to physicians, the most important element in an SOPPC EHR is the system’s ability to generate a medication plan from the view of the medication.

“The most important thing for me of course is the medication plan. It has to work one hundred percent. And that is also a document that we hand to the families, […] so that is important.”

(I_SOPPC_Physician_03:11)

“Then the order, as I said. Somehow it happens that at some point the laxative is at the top [of the list; D.M.] and the strong analgesic in the middle. We would like to give out medication plans that are clearly arranged for the family. […] It would also be good if you could pull it by hand. […] That would be a help.”

(I_SOPPC_Physician_03:19)

In addition, they desired a better transparency of the administration times, for example, facilitated by a consistent structure.

“Also, the different times could be clearer. It is different with adults. There is morning, noon, evening and maybe night. We sometimes have ten different times. And then it becomes confusing.”

(FG_SOPPC_Nurse_01:156)

“Most of the patients don’t have just one drug, but rather 15 to 20. Then you are quite busy already. Somehow finding a consistent structure, […] I think it’s important. So that it is somehow a little bit better specified; […] first active ingredient, then application form, […] concentration or similar.”

(I_SOPPC_Physician_02:41)

To support the families in their child’s medication administration, information on the application method is necessary.

“Yes, there are 13 drops, three fixed administrations; that is three times 13 drops. Because that is what is important for the mother, who gives drops, not milligrams […]”.

(I_SOPPC_Physician_02:37)

In terms of usability, participants requested confirmation prompts, especially when deleting a medication.

“And when I stop, it looks like this. That’s not very good either. If I delete it now, there will be no confirmation.”

(I_SOPPC_Physician_02:41)

Several participants expressed dissatisfaction related to the documentation of infusion pumps.

“Especially with children it is important to determine in how much volume it is administered. Sometimes ten milligrams are in five milliliters, but you can also have it in a hundred milliliters and that sometimes makes a difference for children. […] So i.v. or per os or in the buttocks and then at what times, […] what certain running speed. […] What the doctors […] strive for is the dose and that means the mass of the substance that the child should receive per kilo or per square meter [of the body surface; D.M.]. The most common is per kilo, but there are also situations where this is related to the body surface. And you can’t enter that here.”

(I_SOPPC_Physician_04:26)

SOPPC participants also brought up the need for documentation of on-demand medication administered by the child’s family members.

“It would also be nice if you had something about on-demand medication, where with the individual drugs you can say ‘it has been administered three times during the last 24 hours’, or ‘not at all’, or something. […] Of course, you can also indicate it in the free text, but then you have to search again to see if that was somehow an issue.”

(I_SOPPC_Physician_03:19)

Besides a medication list, PPCU and SOPPC physicians emphasized the value of dosing or nutrition calculators that take infant conditions into account.

“[…] in this unit, there are patients who weigh 12 kilos at the age of 18”

(FG_PPCU_Physician_01:653)

“Actually, you would like to have a system that supports you in calculating. Those are quite simple calculations; everyone can do them if they have time and are relaxed. But then you get three, four in a row. If you do this under time pressure or if you are stressed […], then mistakes happen quite regularly. […] And if you use software that somehow maps the medication process, then it is extremely desirable that you have a calculation aid.”

(I_SOPPC_Physicians_04:28)

#### 3.4.6. Percentile Calculator

From the PPCU nurses’ and SOPPC physicians’ perspectives, the inclusion of a percentile calculator that displays the percentiles could reduce documentation efforts.

“That would also be good if it could be done automatically. You enter the length and size and it automatically inserts this into the percentile curve.”

(FG_PPCU_Nurse_02:179)

“There are things like percentiles, […] in other words, peculiarities that software developers never think of in children. […] to see are they properly nourished? Are they growing normally?”

(I_SOPPC_Physician_04:100)

#### 3.4.7. Specific Care Tools

An EHR for PPC should, from SOPPC participants’ perspectives, include specific care tools that enable structured and plausible documentation concerning specific care issues like ventilation, tracheostoma care, secretion management, or wound care.

“[…] for example, the breathing rate and oxygen saturation are queried, but it does not appear anywhere how much oxygen the patient currently needs and is being given. And that would be nice to be able to do somehow, put a tick next to the vital signs or something, whether it is a ventilated patient or not […]. Otherwise, such vital signs are sometimes not that informative.”

(I_SOPPC_Nurse_03:35)

“Right now, yes, we have to look for a lot in the running text, so to speak. Like when colleague XY […] was with the family, then I have to look for when was she with the family and what did she notice, right? Did the stoma look good at that particular moment, or was there a high need for aspiration or not? And was the secretion yellowish because perhaps somehow there is an infection going on? Yes, something like that. Also for chronic wounds.”

(I_SOPPC_Nurse_03:67-69)

Moreover, nurses wished for an input screen for the Nursing Care Plan (NCP).

“Like I said, we are missing an input screen for the NCP. Right now, the way we do it is we write an NCP as a (word processing program) document and then of course you have the option of uploading that, which is good, but of course it would be nice if that was a direct feature within the system.”

(I_SOPPC_Nurse_02:45)

#### 3.4.8. Psychosocial Tool

PPCU and SOPPC psychosocial participants further expressed the need for a psychosocial tool to specifically document psychosocial issues, which could be subdivided by patient and family. This could also comprise a Psychosocial Care Plan.

“I think that would be very good, then you have everything in one place that is psychosocial.”

(FG_PPCU_Psychosocial_01:131)

#### 3.4.9. Home Visit Tool

According to the participants, the documentation of home visits is a central element in SOPPC. A home visit tool organized according to the typical process and including specific pediatric issues could reduce the documentation efforts and facilitate a home visit routine.

“But the documentation of symptoms also does not provide what is needed in pediatric care. We document an incredible amount in the comment field again in free text. […] I don’t know […] because people like to write a lot anyway […] or if the tools are missing where you can document so it’s a bit more standardized […] But most of it is still done in the comment field. And I think that’s always a sign that the other categories don’t allow that […] or that it’s not adapted.”

(I_SOPPC_Physician_03:3-5)

“In other words, that the structure can be used to map the different levels that are addressed during a home visit. And then you could also do certain things, if you knew, okay, they are now in this section, then maybe you think about them.”

(I_SOPPC_Physician_03:32)

Among other things, the tool should include administrative data (e.g., place, time, family members, and employees present); the rationale for the home visit; a list of distressing symptoms; a physical examination section that includes pediatric parameters; and a section for psychosocial or sociolegal issues.

## 4. Discussion

This study revealed the strengths and weaknesses concerning the current documentation system of one inpatient pediatric palliative care (PPC) team (documenting in a paper record) and three outpatient PPC teams (using an electronic health record (EHR) for adult palliative care). Expectations and concerns regarding electronic documentation in general were explored. Moreover, several demands of an EHR from the perspective of the various professions involved in inpatient and outpatient PPC settings were revealed. Many of these needs can be considered relevant to an EHR in pediatrics in general and correspond to the recommendations by the American Academy of Pediatrics (2020) [3].

Consistent with other publications, participants considered several benefits of electronic documentation, such as having all the data available for the entire treatment period, the simultaneous use of the EHR by multiple providers, or being able to access data from different locations [3,25]. However, these advantages also include special requirements regarding data security to avoid the risk of privacy disclosure by improper authorization and the abuse of data [3,25,26]. Various data protection mechanisms are already used in EHRs to ensure the three fundamental security goals of confidentiality, integrity, and availability [25]. Confidentiality refers to the fact that the data can only be viewed by authorized persons [25,26]. Integrity concerns the accuracy of the data. Additionally, the documentation of the data must be auditable over the entire period of use of the file. Availability implies accessibility by an authorized person [25]. In terms of accessibility, however, it should be ensured that authorization mechanisms are practicable to have quick access to the necessary patient information, especially in crisis situations.

Besides data security, the interoperability of EHR contents is mandatory, as PPC is characterized by frequent changes between the outpatient and inpatient settings [17]. Various providers are involved in the care process so that information is circulated in a timely manner [11,27,28]. However, this demand is more complex than it seems due to heterogeneous interoperability standards [29].

Shared provider documentation is discussed as a requirement for EHR in pediatrics in general [3]. As PPC is characterized by a multi- and interprofessional care approach [11], an EHR should enable the relevant information to be merged from different professions. Nevertheless, profession-specific documentation remains necessary, as indicated by the required specialized care and psychosocial tools.

Participants from both settings strongly expressed the need for clarity concerning the documentation and retrieval of relevant information. Due to the often long-term duration of care for PPC patients and the associated increase in data volume, users could be confronted with information overload and become unable to retrieve important information quickly [3]. Therefore, one important finding was the desire for concise data visualization, such as via customizable views.

**Figure 1:**
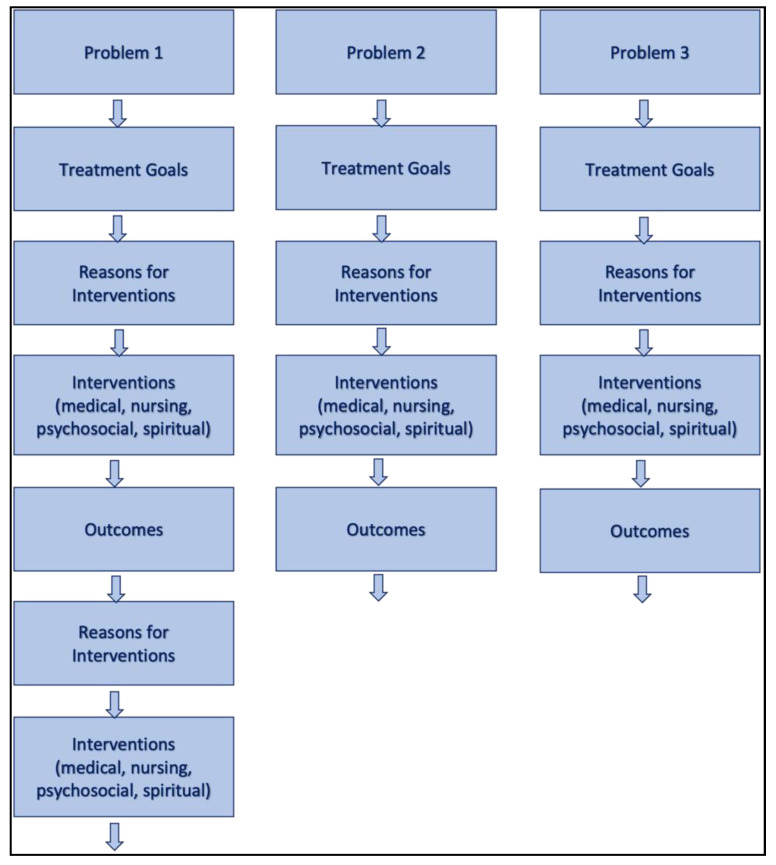
Exemplary scheme for the clear electronic health record (EHR) documentation of a treatment process in pediatric palliative care (PPC).

Another important element to facilitate an information overview is the pronounced need for a treatment process that includes the main problems, treatment goals, interventions, and reasons for the decisions and outcomes. As PPC patients “often face a broad spectrum of interrelated symptoms” [15] (p. 2), and because they are supported for many years, such a treatment process needs to be clearly structured to meet the requirements of clarity. An example of such a process scheme derived from the results is shown in Figure 1.

In addition, both the inpatient and outpatient PPC teams mentioned a patient chart to monitor, among other things, symptom progression. The identification and observation of distressing symptoms are necessary for symptom control [15]. However, this is often complicated by the fact that many PPC patients experience severe neurological impairment and are nonverbal [12,15,17]. To address these circumstances, PPC is based on the biopsychosocial–spiritual care approach, which incorporates body, mind, relationship, and spiritual aspects [30]. A patient chart in PPC should be developed in accordance with this approach [31] and include, for example, vital signs, medications, behavior observations, or therapeutic approaches. Here, it should be possible to represent the setting’s multi-professionality, as well. In order to ensure the clearest possible presentation, it should be feasible to transform different parameters into a visual context.

In contrast to the inpatient setting, a patient’s continuous observation and documentation is not possible in an outpatient setting. Documentation in a patient chart for specialized outpatient PPC teams (SOPPC) could therefore be intermittent/situational and be based on, for instance, home visits or phone calls with patients’ relatives.

Participating physicians highlighted the importance of a medication summary. As PPC patients receive many medications [12], a medication summary could be structured by application form or indication. Information on active ingredients and product names should be obligatory. Relevant information concerning the application form, such as infusion pumps, should be included. The need for the improved recording of medication is underlined by other authors, as it has been shown that drug treatments are insufficiently documented in some medical records of pediatric patients [3,32,33].

Participants also mentioned dosage aid calculations as relevant tools to support the care and safety of patients. The demand for such a tool based on age, weight, body surface, and glomerular filtration rate is supported by the findings of Roedle et al. (2019) [7]. Ratwani et al. (2018) revealed an improper dose as the most common medication error [2]. As pediatric patients are uniquely reliant on the usability of an EHR [2], strong efforts should be undertaken to secure the usability of dosage aid calculators. Moreover, features like drug–drug interaction alerts, information about adverse effects, pharmacokinetics, and pharmacodynamics could increase the quality of the medication prescription [7]. Due to widespread off-label use in pediatrics [33] and presumably in PPC [34], comprehensive- and evidence-based information was demanded [7].

A basic assessment should include the relevant parameters in PPC. Results show that the relevant data for children like social background or abilities (especially important for nonverbal children) cannot be mapped in a basic assessment for adult palliative care. This indicates the general necessity that the contents should fit the special area or sector in which they are used and that the documentation needs in pediatrics differ from those in adult care [10,31].

Finally, participants require EHRs to be adaptable and flexible to emerging needs. Participants already working with an EHR for adults, and who had previously voiced concerns, disclosed that the changes were not implemented in a consistent manner. This corresponds to the findings of Martikainen et al. (2020) that nurses and physicians, although willing to participate in the development of electronic health systems, could not influence their development to the capacity they preferred [21]. Another study, assessing Finnish developers’ viewpoints towards user participation, showed that “both physicians and developers seem to be ‘willing but not able’ to collaborate with each other” [35] (p. 198). Problems in collaboration might result from several breakdowns in the information flow concerning user feedback and that developers often collaborate with customer representatives who are not end users [35]. Perhaps the degree of flexibility and adaptability of EHRs to user demands should be a feature policymakers take into account while implementing specialized EHR systems [31].

It can be assumed that this study does not indicate all the relevant demands towards an EHR in PPC, as we included just one PPCU and three SOPPC teams in the study sample. In addition, the interpretation should take into account that these requirements and preferences relate to the German healthcare system and may differ from other application contexts in other countries. Moreover, some participants from the inpatient setting who had no prior experience with electronic documentation noted that they had difficulty abstracting from the accustomed structure of the paper record to an electronic system. Furthermore, it appears that the participants did not mention aspects that they take for granted, such as admission date. This shows that the survey of user needs for an EHR for PPC cannot be limited to an initial inquiry but, rather, that further steps are required to consolidate what needs should be met, ideally including more inpatient and outpatient teams.

This study provided a first insight into the requirements and preferences concerning an EHR for the specialized inpatient and outpatient settings of PPC. In principle, documentation in an electronic format seems to be ideal for the continuity and traceability of care in this patient population. Overall, the conclusion can be drawn that, even in such a highly specialized setting as PPC, there are needs that are comparable to less-specialized disciplines and pediatrics itself. However, individual specifications and adaptations are necessary for this particular setting to ensure the utmost usability and patient safety. Thus, this study provided some initial guidance for adapting existing EHRs or selecting appropriate EHRs for the PPC setting. Since the present findings and a review of the current literature show that usability often depends on such details, future research and, especially, the participation of users in the development and implementation process of an EHR for PPC are warranted.

## Figures and Tables

**Table 1:** Characteristics of inpatient (*N* = 23) and outpatient (*N* = 11) professionals.

	Inpatient ^a^ *n* (%)	Outpatient ^b^ *n* (%)
Sex		
Female	15 (78.9)	6 (66.7)
Male	4 (21.1)	3 (33.3)
Age in years (SD)	42.9 (11.1)	47.6 (6.9)
Profession		
Nurse	6 (31.6)	4 (44.4)
Physician	6 (31.6)	5 (55.6)
Psychosocial	5 (26.3)	
Secretary	2 (10.5)	
Years of work experience		
0–10	7 (36.8)	
10–20	4 (21.1)	3 (33.3)
20–30	4 (21.1)	5 (55.6)
>30	4 (21.1)	1 (11.1)
Years of experience in current position		
0–4	10 (52.6)	3 (33.3)
4–8	2 (10.5)	2 (22.2)
8–12	4 (21.1)	3 (33.3)
>12	3 (15.8)	1 (11.1)
Experience in professional use of EHRs	7 (36.8)	9 (100)
Years of experience in professional use of EHRs ^c^		
0–4	2 (10.5)	1 (11.1)
4–8	3 (15.8)	2 (22.2)
8–12	1 (5.3)	2 (22.2)
>12	1 (5.3)	2 (22.2)

^a^*n* = 19; (*n* = 4 nurse characteristics missing due to the nonreturn of the questionnaire). ^b^ *n* = 9; (*n* = 2 psychosocial characteristics missing due to the nonreturn of the questionnaire). ^c^ This question was answered by *n* = 7 participants from the outpatient setting (*n* = 2 missing). EHRs: electronic health records.

**Table 2:** General requirements for an electronic health record (EHR).

Subcategories of General Requirements	Specifications
3.3.1 Different data entry options	• Speech recognition • ePens • Templates (discrete data, free text)
3.3.2 Clarity	• Individualizable views • Scalability • Filter function
3.3.3 Integration of multimedia files	• Photo files • Audio files • Video files
3.3.4 Interoperability	• Data transfer between providers involved in the patient’s care
3.3.5 Multi-professional documentation	• Integrative documentation of involved professions
3.3.6 Adaptability	• Customizable contents • Flexibility to new requirements

**Table 3:** Content requirements for an EHR.

Subcategories of Content Requirements	Specific Contents	
3.4.1 Individual page with patient’s core information	• Patient’s core information • Medical Orders for life-sustaining treatment • Diagnoses • Admission reason	
3.4.2 Basic Assessment Tool	Information about • Social background • Social law and care benefits • Abilities (e.g., linguistic abilities) • Degree of disability • Patient’s provider network • Educational institutions	
3.4.3 Treatment Process	Comprehensible process documentation including • Patient’s main symptoms/issues • Interventions • Outcome	
3.4.4 Patient Chart	Inpatient progress documentation • Vital signs • Documentation of symptom observation • Medication • Positioning	Outpatient progress documentation • Vital signs • Documentation of symptom observation • Nursing reports • Comments
3.4.5 Medication Tool	• Medication chart • Calculator for dosages	
3.4.6 Percentile Calculator	• Automatically generated growth charts • Percentile curves	
3.4.7 Specific Care Tools	Templates for • Ventilation documentation • Tracheostoma care • Secretion management • Wound care • Nursing care plan	
3.4.8 Psychosocial Tool	Template for • Psychosocial documentation subdivided by patients and family • Psychosocial care planning	
3.5.9 Home Visit Tool ^a^	Template should map home visit routine and include • Administrative data • Rationale for the home visit • List of distressing symptoms • Physical examination section • Psychosocial and socio-legal issues	

^a^ Only for specialized outpatient pediatric palliative care (SOPPC) teams.

## Data Availability

The corresponding datasets of this study are available from the corresponding author upon reasonable request.
